# Triple Therapy with First Generation Protease Inhibitors for Hepatitis C Markedly Impairs Function of Neutrophil Granulocytes

**DOI:** 10.1371/journal.pone.0150299

**Published:** 2016-03-03

**Authors:** Walter Spindelboeck, Angela Horvath, Monika Tawdrous, Bianca Schmerböck, Gabriele Zettel, Andreas Posch, Andrea Streit, Petra Jurse, Sandra Lemesch, Martin Horn, Gerit Wuensch, Philipp Stiegler, Rudolf E. Stauber, Bettina Leber, Vanessa Stadlbauer

**Affiliations:** 1 Department of Internal Medicine, Division of Gastroenterology and Hepatology, Medical University of Graz, Graz, Austria; 2 Department of Surgery, Division of Transplantation Surgery, Medical University of Graz, Graz, Austria; 3 Institute for Medical Informatics, Statistics and Documentation, Medical University of Graz, Graz, Austria; Clínica Universidad de Navarra, SPAIN

## Abstract

First-generation HCV protease inhibitors represent a milestone in antiviral therapy for chronic hepatitis C infection (CHC), but substantially increased rates of viral clearance are offset by increased rates of infection and infection-associated deaths, especially of patients with advanced liver disease. We aimed to assess whether first generation protease inhibitors interfere with neutrophil function. We included 108 consecutive, retrospective CHC patients and 44 consecutive, prospective CHC patients who were treated with peginterferon and ribavirin with or without protease inhibitors according to the guidelines in the period of November 2012 to June 2015. 33 healthy volunteers served as controls. Infection data were evaluated in all patients. Neutrophil phagocytosis, oxidative burst, elastase and diamine oxidase levels during 12 weeks of triple (n = 23) or dual therapy (n = 21) were studied in the prospective part. In the retro- and prospective cohorts patients experiencing clinically relevant infections were significantly more frequent during protease inhibitor therapy (31% and 26%) than during therapy with peginterferon and ribavirin (13% and 0%). Neutrophil phagocytosis decreased to 40% of baseline with addition of protease inhibitors to P/R but recovered 6 months after end of treatment. Protease inhibitors also seemed to reduce serum elastase levels but did not impact on gut permeability. Impaired neutrophil function during triple therapy with first generation HCV protease inhibitors may explain the high infection rate associated to these treatments and be of relevance for treatment success and patient survival.

**Trial Registration:**

ClinicalTrials.gov NCT02545400

ClinicalTrials.gov NCT02545335

## Introduction

Chronic hepatitis C infection (CHC) is a major health problem that affects more than 185 million persons worldwide and can lead to liver related mortality via liver cirrhosis or hepatocellular carcinoma [1]. Antiviral therapy has evolved during the past 25 years from interferon as standard treatment, over the combination with ribavirin and pegylated interferon (P/R) to the addition of protease inhibitors to P/R and finally to interferon-free regimens. [2, 3]. Between 2012 and 2014 the standard therapy for CHC genotype 1 patients without cirrhosis consisted of ribavirin and pegylated interferon (P/R) in combination with boceprevir (BOC) or telaprevir (TPV), which are direct acting antivirals and represent the first generation of protease inhibitors [4, 5].

Triple therapy for CHC has been reported to be associated with a quantitative and qualitative increase in treatment-related (serious) adverse events compared to the former standard therapy without protease inhibitors. Reports have accumulated of serious infectious complications during triple therapy with considerable morbidity and mortality, especially in patients with acquired immune deficiencies like liver cirrhosis.[6–9] The mechanisms of this increased susceptibility to infections remain unclear, but BOC and TPV are known to inhibit neutrophil elastase activity in vitro [10–12].

Accordingly, this study aimed to analyse infections that developed in our CHC outpatients during therapy and to prospectively characterize neutrophil function in patients undergoing CHC triple therapy in comparison to dual therapy with peginterferon and ribavirin to elucidate possible mechanisms of protease inhibitor associated infections.

## Patients and Methods

The study consisted of two parts: first, in a retrospective phase (NCT02545400), we evaluated and compared the incidence and severity of infections in patients treated for CHC between January 2011 and June 2013 with P/R, with or without BOC or TPV. Patients’ charts were reviewed and clinically relevant infections were recorded. A clinically relevant infection is given if either clinical, radiological, and/or laboratory evidence is found and anti-infective medication (topical, oral or intravenously) and/or hospitalization is necessary. Severity of clinically relevant infections was graded according to the CTCAE criteria (Version 4.03, June 14, 2010). Grade 2 infections require local or minimal intervention; oral or local therapy (e.g., antibiotic, antifungal, antiviral); are limiting age-appropriate instrumental activities of daily life; Grade 3 infections require intravenous antibiotic, antifungal, or antiviral therapy and/or radiologic or surgical interventions; are severe or medically significant but not immediately life-threatening; require hospitalization or prolongation of hospitalization; disabling; limiting self-care activities of daily life; Grade 4 infections have life-threatening consequences where urgent intervention is indicated; and Grade 5 represents a fatal outcome. Grade 1 infections (localized infections requiring local therapy only) were not considered as clinically relevant. The semicolon indicates “or” within the grade. In addition, we analyzed the week of therapy in which infections occurred (week of infection), if patients were hospitalized unplanned for more than 24 hours (hospitalization), and if hepatitis C therapy was discontinued (discontinuation).

In the second, prospective phase (NCT02545335), consecutive patients undergoing therapy for CHC were included. Inclusion criteria were a planned antiviral therapy with P/R with or without BOC or TPV. Patients with immunosuppressive medication, liver cirrhosis, active infection at baseline or treatment with antibiotics within the preceding two weeks were excluded. Patients were treated according to the guidelines that were in place at the time of the study (EASL clinical practice guidelines 2013 and Expert Opinion on Boceprevir- and Telaprevir-Based Triple Therapies of Chronic Hepatitis C 2012). All medications were prescribed according to the package insert in both study parts; in brief, all patients started therapy with P/R and telaprevir was added upon baseline (TPV group), whereas boceprevir was introduced after a four-week long lead-in period with P/R (BOC group). Patients receiving BOC or TPV therapies were never switched to the other regimen. All genotype 1 patients were considered for triple therapy. Treatment-naïve genotype 1 patients with baseline features predicting a high likelihood of rapid virological response and sustained virological response (no fibrosis, low viral load, favourable IL28B genotype) and Genotype 2–3 patients were treated with dual therapy [13, 14].

Both studies are observational; they were registered at clinicaltrials.gov after recruitment has started, since it was not mandatory to register the studies beforehand. Patients received medical treatment according to the approval regulations of the respective drugs and gave written informed consent before study entry. The study conformed to the Declaration of Helsinki. All procedures involving human subjects were approved by the Institutional Review Board of the Medical University of Graz (25–006 ex 12/13 and 25–556 ex 12/13).

Both parts of the study were conducted at the Liver Outpatient Clinics, Department of Internal Medicine, Medical University of Graz, Austria.

Blood samples were taken at baseline (base) and after 4 (4w) and 12 (12w) weeks of therapy and six months after end of therapy (follow up) to assess sustained virological response (SVR). Clinical and biochemical data were recorded.

For comparison 33 age and sex matched healthy controls were included. These subjects had no evidence of liver disease or any other acute or chronic disease and did not take any medication.

### Neutrophil function

The Phagoburst® kit (Glycotope, Heidelberg, Germany) was used as directed by the manufacturer to determine the percentage of neutrophils that produced reactive oxidants with or without stimulation by flow cytometric analysis. A forward-side scatter gate was set on neutrophils and 10,000 neutrophils have been recorded. Data are presented as percentage of bursting neutrophils (FITC positive). An intra-assay precision for percentage of oxidizing cells is given with 0.1% CV by the supplier.

The Phagotest® (Glycotope, Heidelberg, Germany) was used as directed by the manufacturer to measure phagocytosis by flow cytometric analysis using FITC-labelled opsonized *E*. *coli* bacteria. A forward-side scatter gate was set on neutrophils and 10,000 neutrophils have been recorded. Percentage of neutrophils that showed no phagocytic activity was recorded (inactive neutrophils). The phagocytic capacity was calculated by weighing the geometric mean of fluorescence intensity (GMFI) with the percentage of low and high phagocytizing neutrophils. To overcome batch variations all values are presented as *n*-fold change of healthy controls determined by the corresponding batch of bacteria. An intra-assay precision for percentage of phagocytizing cells and GMFI is given with 0.2% CV and 1.5% CV by the supplier.

Flow cytometric analysis was done with an LSRII cytometer in combination with FACS Diva 6.2 software (BD Bioscience, Heidelberg, Germany). Quality control was carried out daily by using CS&T beads.

### PMN elastase

PMN elastase protein levels in heparinized plasma samples were quantified with a ready-to-use solid-phase sandwich ELISA (eBioscience, Vienna, Austria) according to the manufacturer’s instructions. Determinations were done in duplicates and values with a coefficient of variation of <20% were considered trustworthy. PMN elastase protein levels determined were referred to neutrophil count and are given as ng elastase per million neutrophils for analysis.

### Diamine oxidase

A ready-to-use solid-phase sandwich ELISA (Immundiagnostik AG, Bensheim, Germany) was used as directed by the manufacturer to detect diamine oxidase (DAO) in serum samples. Determinations were done in duplicates and values with CV<20% were considered trustworthy.

### Endotoxin

Serum levels of endotoxin were assessed using HEK-Blue™ LPS Detection Kit (Invivogen, San Diego, USA) with adapted protocol. In brief, cells were cultured in 24-well plates (5x10^4^cells/well). After 24 hours medium was discarded and replaced with samples/endotoxin standards and detection medium. Cells are incubated for 21 hours at 37°C and colour intensity is measured at a wave length of 650nm. Only optical density exceeding endogenous alkaline phosphatase activity was considered positive.

### Statistics

Data were analyzed with SPSS 21. Between group differences of categorical variables were assessed by Fisher’s exact test or McNemar test, for unpaired and paired data, respectively. To compare more than two groups pairwise multiple testing with Bonferroni correction was applied. Between group differences of continuous variables were assessed by non-parametric methods: the Mann-Whitney/Wilcoxon signed rank tests and Kruskal-Wallis/Friedman tests for unpaired/paired data to compare two or more groups, respectively. Multiple tests were corrected using Bonferroni correction. Data are given as median (Q1; Q3) unless otherwise stated. All tests were performed at a 5% significance level.

## Results

### Retrospective incidence and type of infections during hepatitis C therapy

Study groups were comparable with respect to age, gender and pre-treatment status. Patients undergoing P/R treatment (n = 47) had significantly fewer infections than those with BOC or TPV therapy (n = 61). In total, six patients (13%) developed clinically significant infections during P/R and 19 patients (31%) during BOC or TPV therapy (p = 0.045), amounting to 7 vs. 23 infections, respectively. Infections developing during P/R treatment led less often to hospitalization (2% and 8% respectively, p = 0.027) or treatment discontinuation (0% and 10% respectively, p = 0.044) than did triple therapy. There were no significant differences in number of infections or severity estimated by CTCAE score between BOC and TPV treated patients; however, with TPV, infections occurred earlier in the treatment course than with BOC. The median weeks of occurrence were 9 and 17, respectively. There were no treatment-related deaths. Details are given in Table 1 (see S1 Table for details on infectious events).

**Table 1:** Patient and infection characteristics for 108 retrospectively analysed patients during hepatitis C therapy.

	TPV	BOC	P/R
**n (m/f)**	26 (12/14)	35 (19/16)	47 (31/16)
**median age (Q1; Q3)**	56 (53; 63)	49 (43; 55)	46 (37; 54)
**therapy naïve**	12 (46%)	18 (51%)	32 (68%)
**Treatment duration in weeks median (Q1; Q3)**	24 (18;25)	28 (21;47)	24 (23;47)
**Clinically relevant infections**	7	16	7
**Patients with clinically relevant infections**	7 (27%)	12 (34%)^2^	6 (13%)
**discontinuation**	4 (15%)^2^	2 (6%)	0 (0%)
**hospitalization**	3 (12%)^2^	2 (6%)	1 (2%)
**week of infection (median)**	9	17	13
**CTCAE (2/3/4)**	3/4/0	7/8/1	5/1/0
**SVR**	13 (50%)	16 (46%)	28 (60%)
**Fibrosis stage**^§^ **(no-mild/significant-severe/cirrhosis/no result)**	4/12/6/4^2^	13/11/7/4	20/8/3/16
**ALT (U/l)**	62 (45;133)	56 (47;99)	80 (38;126)
**neutrophil count (x10**^**6**^**/ml)**	3.7 (2.7;4.3)	3.2 (2.5;3.9)	3.5 (3.0;4.7)
**platelet count (x10**^**6**^**/ml)**	176 (145;255)	228 (151;245)	217 (197;260)
**Albumin (g/dl)**	4.2 (3.1;4.5)	4.3 (4.1;4.7)	4.6 (4.4;4.8)
**haemoglobin (g/dl)**	14.5 (14.1; 15.5)	14.6 (13.6; 15.7)	15.2 (13.7; 16.0)
**bilirubin (mg/dl)**	0.5 (0.3; 0.6)	0.4 (0.3; 0.5)	0.4 (0.2; 0.5)

^2^p<0.05 vs P/R; P/R: Dual therapy with peginterferon/ribavirin; TPV: triple therapy with P/R and telaprevir, BOC: triple therapy with P/R and boceprevir

^**§**^ no-mild fibrosis: Metavir 0–1 or Fibroscan® <7.0 kPa; significant-severe fibrosis: Metavir 2–3 or Fibroscan® 7.0–12.5 kPa; cirrhosis: Metavir 4 or Fibroscan® >12.5 kPa; no result: fibrosis was not assessed or the result from Fibroscan ® was not valid

### Prospective patient characteristics and standard laboratory parameters

**Fig 1:**
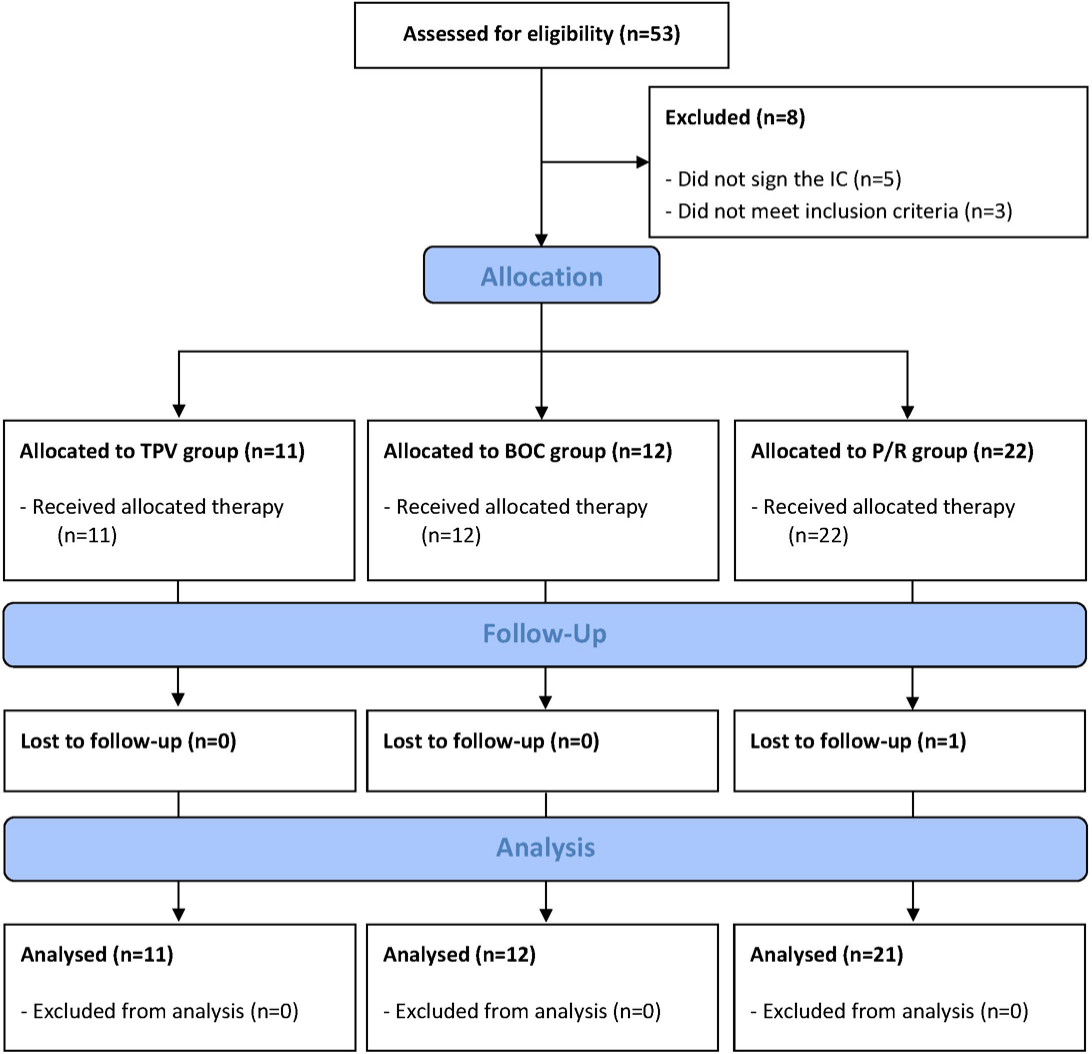
Flow diagram of the study progress.

Fifty-three patients have been screened for the study between November 2012 and March 2014, whereof 5 patients refused to take part and 3 patients did not meet the inclusion criteria. 45 patients receiving either dual- or triple therapy were included into the study. One patient in the P/R group has been lost to follow-up. Forty-four patients were included into the analysis (see Fig 1). Follow-up ended in March 2015. The three study groups were comparable with respect to age and gender. All patients in the TPV (n = 11) and BOC (n = 12) group had genotype 1 infections. In the P/R (n = 21) group the majority of the patients were infected by either genotype 1 (43%) or 3 (38%). In the P/R group significantly more patients (91%) were treatment naïve than in the TPV group (46%; p = 0.005) but there were no differences in pre-treatment status with respect to HCV genotypes. None of the patients in the P/R group developed infections, while 18% in the TPV group and 33% in the BOC group developed clinically relevant infections. In the TPV group 10 (91%), in the BOC group 10 (83%) and in the P/R group 17 (81%) patients did have sustained virological response (SVR) at the end of follow up (see Table 2 for further information).

**Table 2:** Baseline characteristics of 44 patients and 33 controls in the prospective study. Data are presented as median (Q1; Q3) unless otherwise stated.

	TPV	BOC	P/R	Healthy Controls
**n (m/f)**	11 (7/4)	12 (7/5)	21 (15/6)	33 (15/18)
**age**	52 (41; 61)	48 (45; 56)	38 (29; 46)	46 (31; 54)
**therapy naïve**	5 (46%)^1^	8 (67%)	19 (91%)	-
**GT (1/2/3/4)**	11/0/0/0	12/0/0/0	9/1/8/3	-
**clinically relevant infections**	3	4	0	
**patients with clinically relevant infections**	2 (18%)^2^	4 (33%)^1^	0 (0%)	-
**CTCAE (2/3/4)**	3/0/0	2/2/0	0/0/0	-
**SVR**	10 (91%)	10 (83%)	17 (81%)	-
**Fibrosis stage** ^§^ **(no-mild/significant-severe/cirrhosis/no result)**	9/2/0/0	10/2/0/0	19/2/0/0	-
**ALT (U/l)**	48 (40; 68) **	66 (44; 79) **	63 (30; 141) ***	21 (17; 32)
**neutrophil count (x10**^**6**^**/ml)**	3.1 (2.4; 4.0)	3.3 (2.3; 3.8)	3.5 (2.9; 5.4)	4.2 (3.2; 5.0)
**platelet count (x10**^**6**^**/ml)**	262 (211; 299)	193 (153; 239) *	220 (193; 246)	256 (214; 309)
**Albumin (g/dl)**	4.5 (4.5; 5.2)	4.4 (4.2; 4.8)	4.5 (4.4; 4.8)	4.7 (4.5; 5.0)
**haemoglobin (g/dl)**	15.4 (13.8; 16.7)	15.2 (14.1; 15.5)	15.2 (14.2; 15.9)	14.3 (13.7; 15.1)
**bilirubin (mg/dl)**	1.1 (0.7; 1.2) **	0.8 (0.8; 0.9)	0.6 (0.4; 0.8)	0.5 (0.4; 0.6)
**DAO (U/ml)**	8.5 (8.0; 19.5)	10.3 (6.6; 14.3)	16.0 (11.8; 34.5)	15.8 (11.5; 19.9)
**Endotoxin (EU/ml)**	0.0 (0.0; 0.8)	0.0 (0.0; 0.0)	0.0 (0.0; 2.3)	0.0 (0.0; 0.8)

^1^p<0.01 vs P/R

^2^p<0.05 vs P/R

*p<0.05 vs Controls

**p<0.01 vs Controls

***p<0.001 vs Controls

GT: Genotype; P/R: Dual therapy with peginterferon/ribavirin; TPV: triple therapy with P/R and telaprevir, BOC: triple therapy with P/R and boceprevir;; SVR: sustained virological response; ALT: alanine aminotransferase; DAO: Diaminoxidase

^§^ no-mild fibrosis: Metavir 0–1 or Fibroscan® <7.0 kPa; significant-severe fibrosis: Metavir 2–3 or Fibroscan® 7.0–12.5 kPa; cirrhosis: Metavir 4 or Fibroscan® >12.5 kPa; no result: fibrosis was not assessed or the result from Fibroscan ® was not valid.

Alanine aminotransferase (ALT) levels were significantly higher at baseline in all patient groups than in controls, decreased during therapy and reached normal levels at follow-up. Neutrophil counts were slightly lower in patient groups than controls at baseline and decreased equally in all patient groups during therapy. At follow up neutrophil counts reached baseline levels. Haemoglobin levels were within the normal range in patient groups at baseline and similar to control levels. As expected, haemoglobin levels dropped within all patient groups over time but reached baseline levels at follow up. Bilirubin levels were similar to controls at baseline in all groups and did not rise above normal during the study. For baseline details see Table 2, and S1 Table for further information on infectious events.

### Neutrophil function deteriorates during triple therapy

**Fig 2:**
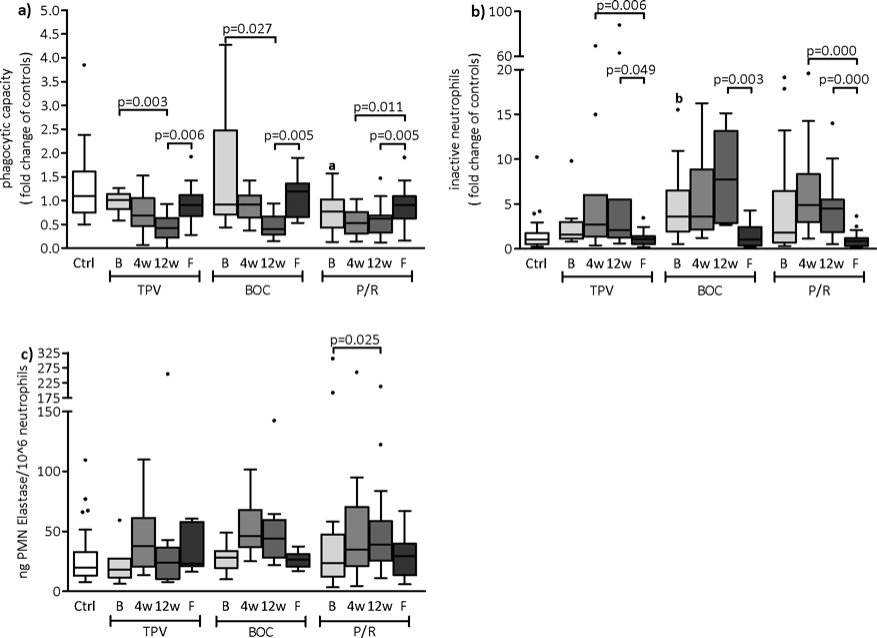
Characteristics of neutrophils in patient groups and healthy controls. Phagocytic capacity of neutrophils (a), proportion of inactive neutrophils (b) and PMN elastase levels per 10^6^neutrophils (c) of patients and controls. Ctrl: healthy controls; TPV: telaprevir group; BOC: boceprevir Group; P/R: Dual therapy with peginterferon/ribavirin; B: baseline; 4w: 4 weeks of therapy; 12w: 12 weeks of therapy; F: follow up a: p = 0.021 vs Ctrl; b: p = 0.010 vs Ctrl.

Phagocytic capacity decreased dramatically during triple therapy with TPV and BOC. In the TPV group the decrease in phagocytic capacity was already evident after 4 weeks: it dropped to 70% of the control value and further decreased to 40% by the end of therapy (p = 0.003 compared to baseline). In the BOC group the phagocytic capacity was maintained at approximately 90% of controls after the first four weeks (lead-in phase) and dropped to 40% at week 12 (p = 0.027 compared to baseline). The P/R group showed no statistically significant decline in phagocytic capacity during therapy (Fig 2A). However, at baseline phagocytic capacity was only 77% of control values (p = 0.021). At follow up phagocytic capacity increased significantly compared to week 12 (TPV: p = 0.006; BOC: p = 0.005; P/R: p = 0.005) and reached almost normal levels in all patient groups (see Fig 2A). HCV genotypes and SVR at follow up did not impact on phagocytic capacity, however the number of patients without SVR is small (n = 7).

In all patient groups, there were more inactive neutrophils at baseline than in controls. This was most prominent in the BOC group (3.6-fold higher than in controls, p = 0.010) and less pronounced in the TPV (1.6-fold) and P/R (1.8-fold) groups. Triple therapy slightly increased the inactive neutrophil count, which was most evident in the BOC group by the end of therapy. However, statistical significance could not be reached and similar patterns could be found in the P/R group. In all patient groups inactive neutrophil counts dropped to levels observed in healthy controls at follow up (Fig 2B). After 12 weeks of therapy, patients infected with genotype 1 had significantly higher levels of inactive neutrophils than those infected with other genotypes (5.4-fold and 2.4-fold of controls, respectively; p = 0.015).

Resting burst increased during therapy significantly in the TPV group (p = 0.008). Priming was similar to controls at baseline in the TPV group but was absent after 4 weeks of therapy (p = 0.002). In the BOC group similar, less pronounced, alterations were detected for resting burst and priming. The P/R group had minimally decreased resting burst at baseline compared to controls, which returned to normal during therapy and dropped to baseline levels at follow up. Priming was low but stable throughout the study in the P/R group.

*E*.*coli* stimulation caused oxidative burst in 93% to 100% of neutrophils in all groups at all time-points. Genotype and SVR at follow up did not impact on any of the burst parameters of neutrophils, however the number of patients without SVR is small (n = 7).

Neutrophil count did not correlate with either phagocytosis capacity or the number of inactive neutrophils or the bursting capacity of neutrophils. The number of patients developing infection during therapy was too small to conduct meaningful statistical analysis concerning neutrophil function. However, patients that developed an infection experienced a steeper decrease in phagocytic capacity over the course of the treatment than patients without infection (76% vs. 45% decrease, respectively). We did not find any trends towards altered neutrophil function in patients with fibrosis compared to those without fibrosis, but again the number of patients with higher grades of fibrosis is too small to allow any conclusions.

### Triple therapy hinders the increase in elastase levels but does not influence gut permeability

Median PMN elastase levels (per 10^6^ neutrophils) were similar to controls in all patient groups at baseline (Ctrl: 19.9 ng/10^6^; TPV: 14.5 ng/10^6^; BOC: 16.2 ng/10^6^; P/R: 21.1 ng/10^6^). PMN elastase levels approximately doubled in the P/R group after 12 weeks of therapy when compared to baseline (39.5 ng/10^6^ neutrophils, p = 0.025) but not in the triple therapy groups (see Fig 2C).

DAO levels showed no differences between patients and controls at baseline levels. Additionally, DAO levels did not increase during therapy and stayed unchanged at follow up. Serum levels of endotoxin did not differ between patients and controls or between patient groups at baseline. Furthermore, no significant changes in endotoxin levels could be observed during therapy (data not shown).

## Discussion

This study shows that first generation protease inhibitors for hepatitis C therapy (BOC and TPV) markedly impair neutrophil phagocytic capacity when administered to non-cirrhotic patients in addition to P/R. This neutrophil dysfunction might be a reason for the higher risk of infection in triple therapy.

The first part of this study confirmed increased rates of clinically significant infections during TPV or BOC treatment for CHC compared to dual therapy with P/R, as previously reported [6–9, 15–18]. Further, only patients undergoing protease inhibitor therapy discontinued treatment due to infectious complications. Hospitalizations for treatment of infections were more frequent in those patients, especially those receiving TPV. The earlier occurrence of infections in the TPV group than in BOC patients goes along with and may be attributable to the lead-in phase for BOC.

In the four phase III studies leading to approval of BOC and TPV in 2011, life threatening infections such as sepsis, bacteraemia or pneumonia were almost exclusively reported in the protease inhibitor arms of the studies, although the absolute numbers were low and no infection-associated deaths were reported [4, 5, 19, 20]. However, the patients were highly selected and there were only small numbers of patients with cirrhosis, a condition known to be associated with a higher risk for infections. The problem of relevant infectious complications was observed during field use of the triple therapy regimens in non-selected populations and when applied to cirrhotics on a larger scale, although preclinical information was available on anti-protease activity in human tissue [6, 7, 11]. In the retrospective part of our study patients treated with TPV had more severe fibrosis than patients in the P/R group, a selection bias caused by treatment guidelines. However, this does not impact on our results, since the majority of infections under triple therapy originated in the BOC group with comparable severity of liver disease than the P/R group.

Patients with cirrhosis were excluded from the prospective part of our study due to known neutrophil dysfunction [21]. A pre-existing neutrophil dysfunction in cirrhotics [22] and the additional impact of protease inhibitors might explain the high incidence of infections and deaths seen in previous studies [6, 7, 23].

Regarding the pathophysiological mechanism that may lead to increased susceptibility to infections during protease inhibitor treatment, the clinical pattern of infections we saw in our BOC and TPV patients suggested an insufficient neutrophil response.

We then prospectively evaluated neutrophil function in consecutive patients undergoing CHC therapy. We found that the phagocytic capacity decreases and the number of inactive neutrophils increases in patients receiving protease inhibitors in addition to P/R. The phagocytic capacity during triple therapy gives a clear picture: in patients receiving BOC, phagocytic capacity is sustained until the end of the P/R lead-in (4 weeks). Phagocytic capacity then drops to levels comparable to TPV patients at week 12 (40% of baseline). In TPV treated patients without lead-in, the decline is already present at week four and progresses downward until week 12. Six months after end of therapy, all neutrophil function parameter returned to normal. Since there were only a few patients (n = 7) without SVR in our study, it is not possible to analyse the influence of SVR on neutrophil function.

The mechanism for this neutrophil dysfunction is not completely understood. Oxidative burst and phagocytosis, two main functions of neutrophils, give us a broad overview of neutrophil functionality. However, it is to be noted, that neutrophil function as assessed in this study show a rather high inter-individual variation and mirrors only the capacity to initiate oxidative burst or engulf *E*.*coli* when directly presented with them. It has been shown that CHC is associated with systemic oxidative stress mediated by a large number of mechanisms [24] which could be an explanation for ATP depletion. Energy depletion was hypothesized to be related to reduced phagocytic function of neutrophils [25]. For this study we cannot draw any further conclusions about recognition, chemotactic, or bacterial killing by neutrophils during CHC therapy, or their reaction to other types of pathogens.

Absolute neutrophil count, as expected, dropped significantly in all treatment groups, however, there was no difference between patients treated with our without protease inhibitors. Since it is well known, that neutropenia during interferon-based HCV therapy is not associated with an increased risk of infection [26–28], this does not explain the increased risk of infections during therapy with TPV or BOC. It is already known that hepatitis C protease inhibitors also inhibit human proteases (eg. elastase, cathepsin B) [11, 12]. Neutrophil serin proteases are key players in bacterial killing and also important in the regulation of innate immune responses [29]. As it is known that elastase activity is inhibited in human plasma which makes the detection of elastase activity and possibly other neutrophil protease activities in serum difficult and unreliable [30], we decided to determine elastase quantitatively to check if cellular production changes during CHC therapy. In our study, despite a parallel drop in the absolute neutrophil count during therapy in all groups, neutrophil elastase levels (per 10^6^ neutrophils) only increased in P/R but not in triple therapy patients. This could imply a reduced bioavailability in BOC and TPV patients. However, our study design does not allow conclusions on the reasons for this lack of increase in elastase levels during triple therapy. Further *in vitro* experiments would be necessary to study the influence of HCV protease inhibitors on different aspects of neutrophil function.

To narrow down the source of this neutrophil dysfunction, we evaluated gut permeability, which has been linked to neutrophil dysfunction in cirrhosis [31], by using serum DAO levels as a marker for gut barrier integrity [32] and endotoxin as a marker of bacterial translocation. DAO expression, however, remained unchanged during triple therapy. In addition, serum endotoxin levels did not change significantly during any type of therapy, suggesting that gut permeability and bacterial translocation is not the driving pathophysiological mechanism in the setting of CHC therapy.

Changes in routine laboratory parameters (ALT, neutrophil count, haemoglobin, etc.) were typical for patients undergoing CHC therapy but did not show any relevant differences in patients treated with or without protease inhibitors, indicating that the clinical and immunological differences did not play any major role.

Our study has some limitations: With newer antiviral drugs now available, the number of patients receiving first generation protease inhibitors has decreased to zero at our centre and elsewhere. Therefore we were not able to recruit a larger number of patients. However, even with those small numbers, the results are clear cut, owing to the magnitude of the effects. To date we do not have data on the impact of the newer antiviral drugs on neutrophil function, but relevant studies are ongoing. However, these studies will not be completely comparable, since in our study presented here, cirrhosis was an exclusion criterion, whereas, due to the policy of our (and many other) health care systems, direct acting antivirals are only accessible for patients with advanced liver damage at the moment.

However, this study clarifies an important mechanism for severe drug-related side effects important for physicians prescribing antiviral therapies. Data from future studies will help to elucidate whether the second generation protease inhibitors also prove to be more selective in vivo, as suggested in vitro. Newly approved protease inhibitors should be monitored for comparable effects.

In conclusion, we observed impaired neutrophil function during triple therapy with first generation protease inhibitors that resolved completely after therapy and may explain the high rate of infections associated with them. Careful patient selection, especially focussing on other risk factors for infection, and close clinical monitoring during therapy is thus warranted in protease inhibitor treatments.

## Supporting Information

S1 TREND Checklist (PDF)

S1 Protocol Study Protocol retrospective study. (PDF)

S2 Protocol Study Protocol prospective study. (PDF)

S1 Table Infectious events in dual and triple therapy patients. (DOCX)
