# Supplementary material for: Triple Therapy with First Generation Protease Inhibitors for Hepatitis C Markedly Impairs Function of Neutrophil Granulocytes
Source: PLoS One. 2016 Mar 3;11(3):e0150299. doi: 10.1371/journal.pone.0150299 (PMC4777445; doi:10.1371/journal.pone.0150299)
Supplement: S1 Protocol — (PDF) [file pone.0150299.s002.pdf]

Quantifizierung der infektiösen Nebenwirkungen unter Dual- und Tripletherapie von Hepatitis C PatientInnen

an der
Klinischen Abteilung für Gastroenterologie und Hepatologie,
im Zeitraum Jänner 2011 bis Juni 2013
Eine retrospektive Studie an 120 PatientInnen

Betreuerin:

Stadlbauer-Köllner, Vanessa, Ass.-Prof. Priv.-Doz. Dr.med.univ.
Klinische Abteilung für Gastroenterologie und Hepatologie,
8036 Graz, Auenbruggerplatz 15
+43 (0)316 385 82282
vanessa.stadlbauer@medunigraz.at

Ko-Betreuer:

Spindelböck, Walter, Dr.med.univ.,
Klinische Abteilung für Gastroenterologie und Hepatologie,
8036 Graz, Auenbruggerplatz 15
+43 (0)316 385 30195
walter.spindelboeck@medunigraz.at

Diplomand:

Horn, Martin, Mag.rer.nat.,
0650/810 39 11
martin.horn@stud.medunigraz.at

Inhaltsverzeichnis

1 Wissenschaftlicher Hintergrund 2
2 Ziel 2
3 PatientInnen 2
4 Zielgrößen (Endpunkte) 2
4.1 Hauptzielgrößen 2
4.2 Nebenzielparameter 2
5 Methodik 2
6 Statistik 3
6.1 Geplante Auswertung 3
6.2 Fallzahlplanung: 3
7 Datenschutz 3
8 Nutzen-Risiko Evaluierung 3
9 Referenzen 3

1 Wissenschaftlicher Hintergrund

Die Anzahl der chronisch mit dem Hepatitis C Virus (HCV) infizierten PatientInnen wird weltweit auf 130 – 210 Millionen, entsprechend ca. 3% der Weltbevölkerung geschätzt. Insgesamt zählen HCV-Infektionen zu den häufigsten Ursachen chronischer Lebererkrankungen weltweit.[1,2]

Die gegenwärtige Standardtherapie für PatientInnen mit chronischer Hepatitis C basiert auf einer Kombination aus Peginterferon und Ribavirin (Dualtherapie), seit wenigen Jahren optional zusätzlich mit einem oralen Proteaseinhibitor (Tripletherapie).[3,4,5] Dabei zeigen PatientInnen mit einer Genotyp 1 HCV Infektion eine höhere Heilungsrate unter Tripletherapie im Vergleich zum dualen Therapieregime[6].

Die Adherence der PatientInnen zum vereinbarten Therapieablauf ist dabei von zentraler Bedeutung. Insbesondere unter Verwendung von Proteaseinhibitoren kann es unter Nichteinhaltung des Therapieregimes zu Resistenzen kommen[4].

Das Auftreten von Nebenwirkungen sowie deren Schweregrad hängt im Allgemeinen vom verwendeten Therapieregime und im Speziellen von Medikamentendosis, Häufigkeit der Einnahme bzw. Verabreichung, sowie dem Applikationsweg ab.[7] Zu den häufigen Nebenwirkungen (betrifft bis zu 95% der PatientInnen) gehören u.a. Fieber, Müdigkeit, Kopfschmerz, Übelkeit, Arthralgien, Depressionen, Hautveränderungen, Neutropenie und Anämie.[8,9,10,11,12]

Im Vergleich der beiden Therapieregime hat sich ein gehäuftes Auftreten von Nebenwirkungen bei der Tripletherapie gezeigt. Insbesondere kommt es zu einem vermehrten Auftreten von Anämie, Neutropenie, Gastrointestinale Symptome, Müdigkeit, Geschmacksstörungen und Hauterscheinungen.[4,9,6]

Es wurden vermehrt infektiöse Nebenwirkungen unter Tripletherapie beobachtet, welche unter der Dualtherapie mit Peginterferon und Ribavirin nicht oder nur sehr selten beobachtet werden.

Die klinische Bedeutung von Nebenwirkungen der Therapie ist eine Reduktion der Lebensqualität der Patienten und ein damit einhergehender Verlust der Adherenz zur antiviralen Therapie.[7] Die Früherkennung und die unmittelbare Therapie der Nebenwirkungen sind somit unter Anderem entscheidende Parameter im Heilungserfolg der antiviralen Therapie.

2 Ziel

Kernaufgabe der Diplomarbeit ist, das Ausmaß an aufgetretenen Nebenwirkungen (insbesondere infektiösen) unter Dual- bzw. Tripletherapie, von PatientInnen welche an der klinischen Abteilung für Gastroenterologie und Hepatologie der Medizinischen Universität Graz behandelt werden, zu quantifizieren.

Dabei ist mit einem gehäuften Auftreten von Nebenwirkungen unter Tripletherapie zu rechnen.

Der theoretische Teil der Diplomarbeit befasst sich mit der Analyse von möglichen Prädiktorparametern für das Auftreten von Nebenwirkungen unter Therapie. Dazu werden die gewonnen Ergebnisse mit einer ausgedenhten Literaturrechereche verglichen.

Da chronische Hepatitis C beide Geschlechter betrifft, ist die angestrebte Zielsetzung für Frauen und Männer gleichermaßen bedeutsam.

3 PatientInnen

Das PatientInnenkollektiv umfasst alle im Zeitraum Jänner 2011 bis Juni 2013 an der Leberambulanz der klinischen Abteilung für Gastroenterologie und Hepatologie der Medizinischen Universität Graz therapierten Hepatitis C PatientInnen, welche nach den aktuellen, von der European Association for the Study of the Liver (EASL, [4]) definierten Behandlungsrichtlinien oder im Rahmen klinischer Studien, mit Dual- (Ribavirin und pegylierten Interferon-Alpha) bzw. Triple-Therapie (zusätzlich entweder Boceprevir oder Telaprevir, Proteasehemmer) behandelt wurden. Das sind in beiden Gruppen nach ersten Erhebungen etwas mehr als 60 Personen.

4 Zielgrößen (Endpunkte)

Folgende Daten werden retrospektiv, über den aufgezeichneten Therapiezeitraum (vor Therapiebeginn, sowie zu den fortlaufenden Kontrollen unter Therapie) erhoben:

- Alter, Gewicht, Körpergröße, Geschlecht

- Vor- und Begleiterkrankungen, insb. Risikofaktoren (z.B.: Alkoholkonsum, Rauchen, Diabetes, Hepatitiden anderer Genese).

- Aktuelle Eigenmedikation, sowie Art, Dosis und Verlauf einer eventuellen Vortherapie.

- Laborparameter, insb. Blutbild, ALT, AST, GGT, AP, Bilirubin, Albumin, PT, INR, Elektrolyte, Creatinin, TSH, Glucose, Harnstoff, Ferritin, ANA, HCV-Genotyp, IL28-Genotyp, HCV-RNA, HIV, HAV-Antikörpter, HBs-Antikörper, HBs-Antigen, HBc-Antikörper.

- Begleiterscheinungen unter Therapie, insb. klinische Nebenwirkungen (z.B.: starke Müdigkeit, Depression, starke Reizbarkeit, Schlafstörungen, Hauterscheinungen, Dyspnoe, Neutropenie, Anämie, Thrombocytopenie, Krämpfe, Bakterielle Infektionen, Autoimmunreaktionen, interstitielle Lungenerkranungen, Neuroretinitis, Knochenmarksaplasie).

4.1 Hauptzielgrößen

Hauptzielgröße ist die Anzahl an aufgetretenen infektiösen Nebenwirkungen unter Dual- bzw. Tripletherapie.

Weitere Nebenwirkungen werden anhand der vorhandenen Literatur definiert [3-14] und sind insbesondere starke Müdigkeit, Depression, starke Reizbarkeit, Schlafstörungen, Hauterscheinungen, Dyspnoe, Neutropenie, Anämie, Thrombocytopenie, Krämpfe, Autoimmunreaktionen, interstitielle Lungenerkranungen, Neuroretinitis, und Knochenmarksaplasie.

4.2 Nebenzielparameter

Nebenzielgrößen sind alle weiteren unter *4. Zielgrößen* gelisteten Parameter, welche routinemäßig im Therapieverlauf erhoben werden.

5 Methodik

Retrospektive Analyse unter Verwendung der elektronischen Krankenakten und der Ambulanzakten der Abteilung für Gastroenterologie und Hepatologie.

6 Statistik

6.1 Geplante Auswertung

Die Auswertung erfolgt mittels deskriptiver Statistik.

Für numerische Daten werden – abhängig von der Verteilung – Mittelwerte und Standardabweichungen oder Mediane und Quartile berechnet.

Kategorische Daten werden als absolute und relative Häufigkeiten dargestellt.

Die Hauptzielgröße wird mittels abhängigem t-Test analysiert.

6.2 Fallzahlplanung:

Wir erwarten ca. 60 Datensätze pro Therapiegruppe (Dual vs. Triple). Da es sich um eine rein explorative Studie handelt wurde auf eine formale Fallzahl/Power-Berechnung verzichtet.

7 Datenschutz

Alle Patienten werden mit einer fortlaufenden Nummer codiert (pseudonymisiert). Die auszuwertenden Daten werden nur mit diesem Code versehen in einer Excel-Tabelle auf einem PC mit Zugriffsbeschränkung an der Abteilung für Gastroenterologie und Hepatologie gespeichert und anschließend ausgewertet.

Nur autorisierte Personen haben Zugriff auf die Originaldaten.

8 Nutzen-Risiko Evaluierung

Die eingeschlossenen Patienten haben keinen direkten Nutzen von der Studie.

Da es sich allerdings um die rein retrospektive Auswertung ihrer Daten handelt ist auch kein Risiko zu erwarten.

Das einzig mögliche Risiko, das Bekanntwerden der sensiblen PatientInnendaten wird durch die Pseudonymisierung und Zugriffsbeschränkung minimiert.

Die Ergebnisse dieser Studie können als Grundlage zur Hypothesengenerierung für weitere Studien dienen.

Referenzen

[1]Lavanchy D., 2009. The global burden of hepatits C. Liver Int, vol. 29:74-81.

[2]World health organisation. Fact sheet on Hepatits C. Published online: www.who.int/mediacentre/factsheets/fs164/en/index.html. Updated July 2013.

[3]The Japan society of hepatology, 2013. Guidelines for the management of hepatitic c virus infection. Hepatlogy Research, vol. 43:1-34.

[4]Yee H.S. Et al, 2012. Update on the Management and Treatment of Hepatitis C Virus Infection: Recommendations from the Department of Veterans Affairs Hepatitis C Resource Center Program and the National Hepatitis C Program Office. Am J Gastroenterol. advance online publication, 24 April 2012; doi:10.1038/ajg.2012.48.

[5]European association for the study of the liver, 2011. EASL clinical practice guidelines: management of hepatitis c virus infection. Journal of Hepatology, vol. 55:245-264.

[6]Chou R., and Hartung D., and Rahman B., and Wassn N., and Cotrall E.B., and Fu R., 2013. Comparative Effectiveness of antiviral treatment for hepatitis C virus infection in adults: a systematic review. Ann Intern Med, vol. 158:114-123.

[7]Poniachik J., 2006. Management of adverse reactions to chronic hepatits C treatment. Annales of Hepatology, vol. 5(1): S67-S68.

[8]Sung H., and Chang M., and Saab S., 2011. Management of Hepatitis C antiviral therapy adverse effects. Curr Hepatits Rep, vol. 10:33-40.

[9]Sherman K.E., 2012. Managing adverse effects and complications in completing treatment for hepatits C virus infection. HCV Treatment Complications, col. 20(4):125-128.

[10]Presescu O., and Streba L.A.-M., and Irimia E., and Streba L., and Mogoanta L., 2012. Adverse effects of peg-Interferon and Ribavirin combined antiviral treatment in a Romanian hepatitis C virus infected cohort. Rom J Morphol Embryol. Vol 53(3):497-502.

[11]Aspinall R.J., and Pockros P.J., 2004. Review article: the management of side-effects during therapy for hepatitis c. Aliment Pharmacol Ther, vol. 20:917-929.

[12]Fried M.W., 2002. Side effects of therapy of hepatits C and their management. Hepatoogy, vol. 36:S237-S244.

[13]U.S.department of health and human services, 2010. Common Terminology Criteria for Adverse Events (CTCAE), Version 4.03, published online: June 2010.

[14]Education Initiative in Gastroenterology, 2003. HCV side effects management handbook. Approved and Accredited by the Society of Gastroenterology Nurses and Associates.
