# Supplementary material for: Triple Therapy with First Generation Protease Inhibitors for Hepatitis C Markedly Impairs Function of Neutrophil Granulocytes
Source: PLoS One. 2016 Mar 3;11(3):e0150299. doi: 10.1371/journal.pone.0150299 (PMC4777445; doi:10.1371/journal.pone.0150299)
Supplement: S2 Protocol — (PDF) [file pone.0150299.s003.pdf]

STUDIENPROTOKOLL

Leukozytenfunktion bei Kombinationstherapien mit Proteaseinhibitoren gegen Chronische Hepatitis C

Principal Investigators:

PD Dr Vanessa Stadlbauer-Köllner
Dr. Walter Spindelböck

Co-Investigators:

Univ Prof Dr. Rudolf Stauber
Dr. Phillip Douschan
Mag. Dr. Bettina Leber
Mag. Angela Horvath
Sandra Lemesch

Inhaltsverzeichnis:

Kurzfassung ... 3
Wissenschaftlicher Hintergrund ... 5
Opportunistische Infektionen ... 5
Interaktion der Proteaseinhibitoren mit dem Immunsystem ... 6
Zielsetzung ... 7
Primäre Hypothese: ... 7
Sekundäre Hypothesen: ... 7
Ein- und Ausschlusskriterien ... 8
Einschlusskriterien: ... 8
Ausschlusskriterien ... 8
Definitionen ... 9
Studiendesign ... 10
Messgrößen und Zeitraum ... 11
Messgrössen: ... 11
Zeitraum: ... 12
Potentielle Störgrößen ... 13
Biometrische Auswertungsverfahren: ... 13
Studienumfang ... 13
Studienorganisation ... 13
Datenschutz ... 13
Patientensicherheit ... 14
Abbruch der Untersuchung: ... 14
Monitoring: ... 14
Publikation ... 14
Referenzen ... 15

Kurzfassung

Protokolldatum	10.02.2014
Titel	Leukozytenfunktion bei Kombinationstherapien mit Proteaseinhibitoren gegen Chronische Hepatitis C
Sponsor	Akademische Studie
Hintergrund	Es konnte unter Dreifachtherapien (Interferon, Ribavirin plus Boceprevir oder Telaprevir) gegen Chronische Hepatitis C (CHC) eine hohe Rate von Infektionen unter Therapie beobachtet werden. Insbesondere fällt eine hohe Rate von opportunistischen Infektionen (zB Pilzerkrankungen, Herpesvirusreaktivierungen) unter diesen Therapien auf.
Hypothese	Die Einnahme von Protease- oder Polymeraseinhibitoren zusätzlich zur Therapie mit PegInterferon und Ribavirin verursacht laborchemisch nachvollziehbare Funktionsstörungen der Leukozyten beim Menschen.
Ziel	Ziel der vorliegenden klinischen Untersuchung ist es, bei Patienten mit CHC, welche sich unter Therapie mit Interferon, Ribavirin und Boceprevir, Telaprevir, Simeprevir oder Sofosbuvir befinden, den Einfluss dieser Tripelkombinationstherapie auf deren Immunsystem, insbesondere auf die Leukozytenfunktion, zu untersuchen.
Studiendesign	Prospektive Beobachtungsstudie
Patientenanzahl	6 Gruppen:<br><br>GRUPPEN 1, 2, 5 und 6:<br>Patienten mit Tripelkombinationstherapie (nach mindestens 4 Wochen Therapie)<br>Gruppe 1: Boceprevir + Ribavirin + PegInterferon<br>Gruppe 2: Telaprevir + Ribavirin + PegInterferon<br>Gruppe 5: Simeprevir + Ribavirin + PegInterferon<br>Gruppe 6: Sofosbuvir + Ribavirin +/- PegInterferon<br><br>GRUPPE 3:<br>Patienten mit Standardtherapie (Peginterferon alpha 2a/b, Ribavirin) gegen Hepatitis C (Kontrollen)

	GRUPPE 4:<br>Gesunde Probanden (Kontrollen)
Zielpopulation	Patienten mit CHC laufender Dual- und Tripelkombinationstherapie.
Studiendauer	Gesamtdauer: 12 Monate<br>Einzelner Patient: 8 Monate
Methoden	Klinische Evaluierung der Patienten hinsichtlich Infektion<br><br>Standardlaborparameter<br><br>Zusätzlich:<br><br>Neutrophilenfunktion:<br>Phagozytose (Phagotest, Glycotope)<br>oxidativer Burst (Phagoburst, Glycotope)<br>bakterielles „killing“<br><br>Konzentration Neutrophiler Elastase<br><br>Funktion Neutrophiler Elastase

Wissenschaftlicher Hintergrund

Rund 130 bis 170 Millionen Personen sind weltweit mit chronischer Hepatitis C infiziert, in Europa wird von rund 15 Millionen Erkrankten ausgegangen [1]. Dabei ist die chronische Hepatitis C (CHC) weltweit einer der häufigsten Gründe für fortgeschrittene Lebererkrankungen wie Leberzirrhose [2]. Spontanremissionen der CHC werden nicht beobachtet.

Aktuelle Therapiekonzepte bestehen aus einer Kombination von Interferon und antiviral wirksamen Nukleotid- und Nukleosidanaloga. Bisher setzte sich die Standardtherapie gegen CHC aus einer Kombination von dem als Prodrug verabreichten Nukleotidanalogon Ribavirin und pegyliertem Interferon alpha 2 zusammen [3]. Es wurden kürzlich mehrere (Zulassungs)studien zu neuen kleinmolekularen antiviral wirksamen Substanzen (virale Proteaseinhibitoren wie Boceprevir und Telaprevir) bei chronischer Hepatitis C des Genotyps 1 abgeschlossen, welche die Heilungsrate durch Kombination mit der Standardtherapie deutlich erhöhen [4,5].

Präliminäre Ergebnisse der Gruppen 1 und 2 deuten stark auf eine relevant eingeschränkte Neutrophilenfunktion unter den o.g. Dreifachtherapien hin.
Im Jahr 2014 werden zwei weitere antiviral wirksame Substanzen (Simeprevir und Sofosbuvir) in Österreich zugelassen. Da für die neuesten Substanzen ähnliche Wirkungsmechanismen postuliert werden, sind auch ähnliche Nebenwirkungen auf die Neutrophilenfunktion zu erwarten. Es soll dies daher prospektiv analysiert werden. Insbesondere ist diese Fragestellung auch im Hinblick auf lebertransplantierte Patienten oder Patienten mit bereits bestehender Leberzirrhose interessant, da bei diesen die gleichzeitig eingenommene Immunsuppression bzw. durch die Grunderkrankung das Infektionsrisiko erhöht.

Opportunistische Infektionen

Es konnte unter diesen Dreifachtherapien (Interferon, Ribavirin plus Boceprevir oder Telaprevir) eine hohe Rate von Infektionen unter Therapie beobachtet werden (Fachinformationen Victrelis und Incivo). Insbesondere fällt eine hohe Rate von opportunistischen Infektionen (zB Pilzerkrankungen, Herpesvirusreaktivierungen) unter diesen Therapien auf.
Es konnte weiters bei einem in unserer Klink in Behandlung stehenden Patienten die Entwicklung einer pulmonalen Nokardiose (opportunistische bakterielle Infektion der Lunge) beobachtet werden [6].

Diese Infektionen sind insofern ungewöhnlich, da unter der bisherigen Standardtherapie (Interferon, Ribavirin) trotz teilweise ausgeprägter Neutropenie die Entwicklung derartiger Infektionen nicht beobachtet wurde. Zudem sind opportunistische Infektionen eher für Patienten unter immunsuppressiver Therapie (Kortison, Cyclosporin A etc.) typisch, da diese Medikamente per definitionem mit dem menschlichen Immunsystem interagieren und dieses unterdrücken sollen.

Interaktion der Proteaseinhibitoren mit dem Immunsystem

Es liegen zur Interaktion von Boceprevir und Telaprevir mit dem menschlichen Immunsystem nur sehr spärliche Daten vor.
Die selektive und hochwirksame Hemmung der humanen Neutrophilen-Elastase durch Boceprevir konnte bereits gezeigt werden [7].

.

.

Neutrophile Granulozyten sind Zellen der unspezifischen Infektionsabwehr, die bei Infektionen ins Gewebe wandern und dort die Erreger (Bakterien und Pilze) phagozytieren und verdauen.

Zielsetzung

Ziel der vorliegenden klinischen Untersuchung ist es, bei Patienten mit CHC, welche sich unter Therapie mit Interferon, Ribavirin und Boceprevir oder Telaprevir befinden, den Einfluss dieser Tripelkombinationstherapie auf deren Immunsystem, insbesondere die Leukozytenfunktion, zu untersuchen.

Primäre Hypothese:

Die Einnahme von Boceprevir oder Telaprevir zusätzlich zur Therapie mit PegInterferon und Ribavirin verursacht Funktionsstörungen der Phagozytose und des bakteriellen „killing“ der neutrophilen Granulozyten beim Menschen

Zudem wird die Konzentration und Funktion der neutrophilen Elastase beeinträchtigt.

Sekundäre Hypothesen:

Die Einnahme von Boceprevir oder Telaprevir zusätzlich zur Therapie mit PegInterferon und Ribavirin führt zu einer erhöhten Rate an klinisch signifikanten Infektionen

Ein- und Ausschlusskriterien

Einschlusskriterien:

- CHC

- Kombinationstherapie mit Peginterferon alpha 2a/b, Ribavirin und Boceprevir (Gruppe 1) ODER Telaprevir (Gruppe 2) ODER Simeprevir (Gruppe 5) ODER Sofosbuvir (Gruppe 6)

- Kombinationstherapie mit Peginterferon alpha 2a/b, Ribavirin (Gruppe 3)

- gesunde Kontrollen ohne Hepatitis C (Gruppe 4)

- Einwilligung des Patienten/Probanden

Ausschlusskriterien

- Infektion zu Therapiebeginn

- Therapie mit Antibiotika während der letzen 2 Wochen

Definitionen

Klinisch signifikante Infektion: Eine Infektion die mittels mikrobiologischer, radiologischer oder laborchemischer Methoden diagnostiziert wird und die zu einer stationären Aufnahme führt oder eine ambulante Therapie notwendig macht.

Studiendesign

Die vorliegende klinische Untersuchung ist eine Beobachtungsstudie.

Es werden Probanden/Patienten in sechs Gruppen zu je 20 Personen untersucht:

GRUPPE 1, 2, 5 und 6:
Patienten unter laufender Tripelkombinationstherapie (nach mindestens 4 Wochen Therapie)
Als Vergleichswerte werden von diesen Patienten Blutproben vor Therapie und/oder nach abgeschlossener Therapie (zB 6 Monate nach Therapieende) untersucht.

GRUPPE 3:
Patienten unter laufender Standardtherapie (Peginterferon alpha 2a/b, Ribavirin) gegen Hepatitis C.
Als Vergleichswerte werden von diesen Patienten Blutproben vor Therapie oder nach abgeschlossener Therapie (zB 6 Monate nach Therapieende) untersucht.

GRUPPE 4:
Gesunde Probanden.
Als gesunde Probanden werden Mitarbeiter der Medizinischen Universität Graz herangezogen. Die Probanden werden durch persönliche Anrede rekrutiert.
Ein Abhängigkeitsverhältnis wird umgangen, indem in erster Linie Personen anderer klinischer Abteilungen rekrutiert werden.

Messgrößen und Zeitraum

Messgrössen:

1. Anamnestische Daten:
Alter, Komorbiditäten (+Dauer), Medikation, Dauer der Hepatitis-C Virusinfektion

2. Physikalischer Status:
Größe, Gewicht, Internistische Statusuntersuchung, Oberbauchsonographie, Fibroscan

3. Laborwertbestimmungen:

Neutrophilenfunktion

Phagozytose (Phagotest, Glycotope)
Für diesen Test werden 100*µ*l heparinisiertes Vollblut mit einer definierten Menge an FITC gelabelten *E. coli* für 10 min bei 37℃ inkubiert. Ein Kontrollröhrche n mit derselben Mischung Vollblut/Bakterien bleibt währenddessen auf Eis. Nach der Inkubationszeit werden eventuell an der Oberfläche der Zellen gebundene Bakterien mit einer Quenching-Lösung behandelt, um falsch positive Fluoreszenzwerte zu verhindern. Nachfolgend werden die Erythrozyten lysiert und die weißen Blutzellen gleichzeitig fixiert. Nach einer 10-minütigen Inkubationszeit auf Eis mit DNA-staining Lösung werden die Zellen am LSRII auf grüne Fluoreszenz (FITC) vermessen. Es werden 10000 neutrophile Granulozyten aufgenommen.

oxidativer Burst (Phagoburst, Glycotope)
Für diesen Test werden 100*µ*l heparinisiertes Vollblut mit einer definierten Menge an *E. coli* für 10 min bei 37℃ inkubiert. Ein Kontrollröhrche n mit für die Kontrolle enthält Vollblut und Waschlösung, die „Priming Kontrolle“ wird mit fMLP Arbeitslösung gemischt und der „high burst Kontrolle“ wird PMA beigesetzt und ebenfalls für 10 min bei 37℃ inkubiert. Nach der Inkubationszeit wird allen Röhrchen eine Substratlösung (wird durch oxidieren grün fluoreszent) beigemengt und es folgt erneut eine 10 minütige Inkubationszeit bei 37℃. Nachfolgend werden die Er ythrozyten lysiert und die weißen Blutzellen gleichzeitig fixiert. Nach einer 10-minütigen Inkubationszeit auf Eis mit DNA-staining Lösung werden die Zellen am LSRII auf grüne Fluoreszenz vermessen. Es werden 10000 neutrophile Granulozyten aufgenommen.

bakterielles „killing“
Aus Vollblut isolierte neutrophile Granulozyten werden 1:1 mit opsonisierten *E. coli* gemischt (Testansatz) und bei 37℃ inkubiert. Als K ontrolle wird eine Probe mit *E. coli* ohne neutrophilen Granulozyten verwendet und ebenfalls bei 37℃ inkubiert. Aus dem Testansatz werden nach 10, 20 und 30 Minuten Inkubation Proben entnommen und sowohl aus den Überständen, als auch aus den aufgeschlossenen Granulozyten Aliquots auf LB-Platten ausplattiert. Vom Kontrollansatz werden vor Beginn der Inkubation und nach 30 Minuten Inkubation bei 37℃ Proben entnommen und ebenfalls

ausplattiert. Nach Inkubation der Platten bei 37℃ über Nacht werden die entstandenen Kolonien ausgezählt und auf die eingesetzte Menge *E. coli* bezogen.

Konzentration Neutrophiler Elastase

Für die Bestimmung dieser Parameter wird ein handelsüblicher ELISA Kit verwendet. Dazu werden Serumproben an Antikörper gecoatete Wells gebunden und danach mit einem HRP konjugierten Antikörper versetzt. Nach diesem Inkubationsschritt wird Substratlösung hinzu gegeben und die Reaktion mit Hilfe einer Stoplösung beendet. Direkt nach der Zugabe der Stoplösung werden die Proben in einem Platereader (450nm) vermessen.

Aktivität neutrophiler Elastase

Dieser Prameter soll mit Hilfe eines handelsüblichen Immunocapture Activity Assay aus Plasmaproben bestimmt werden. Dazu werden die Proben mit einem Capturing Antikörper inkubiert und danach mit einer Substratlösung versetzt. Nach 4h Inkubationszeit werden die Proben in einem Fluoreszenzreader (Ex: 360nm; Em: 440nm) vermessen.

Zeitraum:

Beginn der Rekrutierung der StudienpatientInnen:	sofort nach Erhalt des EK-Votums
Vollständige Inkludierung aller PatienInnen:	+ 6 Monate
Ende der Daten-/Probensammlung	+ 6 Monate
Datenauswertung:	+ 3 Monate

Potentielle Störgrößen

Vortherapie mit Interferon und/oder Ribavirin, Lebensstilfaktoren (Alkoholabusus, Nikotinabusus, ...), Begleitmedikation, Vorerkrankungen

Biometrische Auswertungsverfahren:

1. Deskriptive Datenanalyse

2. Gruppenvergleiche mit nicht-parametrischen Verfahren (Mann-Whitney-U, Kruskal-Wallis Test) bei nicht normalverteilten Stichproben sowie t-Test und ANOVA bei normalverteilten Daten. Test auf Normalverteilung mittels Kolmogorow-Smirnow Test.

Studienumfang

Es wird mit einer Stichprobengröße von je 20 Patienten pro Gruppe gerechnet. Gesamtzahl an Patienten somit 120.

Studienorganisation

Die Organisation und Durchführung der Untersuchung findet an der Klinischen Abteilung für Gastroenterologie und Hepatologie der Universitätsklinik für Innere Medizin Graz statt.

Datenschutz

Patientendaten werden anonymisiert.
Die Originaldaten werden im Referenzzentrum aufbewahrt, Einblick in diese Dokumente haben nur die Studienleiter.

Patientensicherheit

Es ist für die teilnehmenden Patienten mit keinem Auftreten von schwerwiegenden Nebenwirkungen zu rechnen.

Abbruch der Untersuchung:

- Widerruf des Einverständnisses
- Verletzung des Studienprotokolls
- Weitere Umstände, welche die Patientengesundheit bei Fortsetzen der Studienteilnahme gefährden würden

Monitoring:

Es werden detaillierte Aufzeichnungen über die Studienvisiten, Untersuchungsergebnisse, Begleiterkrankungen sowie Nebenwirkungen der antiviralen Medikation geführt.
Die vom Monitor eingesehenen Daten werden von diesem unter Wahrung der Schweigepflicht behandelt.

Publikation

Publikationen der Ergebnisse in wissenschaftlichen Fachjournalen werden angestrebt.
Für die Reihenfolge der Autorenschaft wird folgendes Vorgehen festgelegt:
(Joint-) Erst- und Letztautorenschaft fällt den Studieninitiatoren im Studienzentrum (WS, VS) zu.
Die Reihenfolge der Co-Autorenschaft orientiert sich in erster Linie am Grad der beigetragenen wissenschaftlichen Leistung.

Referenzen

[1] Cornberg M, Razavi HA, Alberti A, Bernasconi E, Buti M, Cooper C, et al. A systematic review of hepatitis C virus epidemiology in Europe, Canada and Israel. Liver Int 2011 Jul;31 Suppl 2:30-60.

[2] Davis GL, Alter MJ, El-Serag H, Poynard T, Jennings LW. Aging of hepatitis C virus (HCV)-infected persons in the United States: a multiple cohort model of HCV prevalence and disease progression. Gastroenterology 2010 Feb;138(2):513-21, 521.e1-6.

[3] Rosen HR. Clinical practice. Chronic hepatitis C infection. N Engl J Med 2011 Jun 23;364(25):2429-2438.

[4] Poordad F, McCone J,Jr, Bacon BR, Bruno S, Manns MP, Sulkowski MS, et al. Boceprevir for untreated chronic HCV genotype 1 infection. N Engl J Med 2011 Mar 31;364(13):1195-1206.

[5] Jacobson IM, McHutchison JG, Dusheiko G, Di Bisceglie AM, Reddy KR, Bzowej NH, et al. Telaprevir for previously untreated chronic hepatitis C virus infection. N Engl J Med 2011 Jun 23;364(25):2405-2416.

[6] Putz-Bankuti et al 2012, unpublished

[7] Njoroge FG, Chen KX, Shih NY, Piwinski JJ. Challenges in modern drug discovery: a case study of boceprevir, an HCV protease inhibitor for the treatment of hepatitis C virus infection. Acc Chem Res. 2008 Jan;41(1):50-9.
