# Supplementary material for: Triple Therapy with First Generation Protease Inhibitors for Hepatitis C Markedly Impairs Function of Neutrophil Granulocytes
Source: PLoS One. 2016 Mar 3;11(3):e0150299. doi: 10.1371/journal.pone.0150299 (PMC4777445; doi:10.1371/journal.pone.0150299)
Supplement: S1 Table — (DOCX) [file pone.0150299.s004.docx]

**S1 Table.** Infectious events in dual and triple therapy patients (some patients had more than 1 infectious event)

| **1) Retrospective:** | |  |
| --- | --- | --- |
| *a) Dual therapy with peginterferon /ribavirin (P/R)* | | grade |
|  | Urinary tract infection | 3 |
|  | Respiratory infection (unspecified) | 2 |
|  | Recurrent bronchitis | 2 |
|  | Bronchitis x 2 | 2 |
|  | Herpes labialis x 2 | 2 |
| *b) P/R + Boceprevir* | | |
|  | Pleural empyema, pulmonary nocardiosis | 4 |
|  | Urinary tract infection | 3 |
|  | Otomycosis x 2 | 3 |
|  | Invasive gastroenteritis | 3 |
|  | Influenza | 3 |
|  | Tonsillitis x 3 | 3 |
|  | Parodontitis x 3 | 2 |
|  | Herpes labialis x 2 | 2 |
|  | Gingivitis | 2 |
|  | Pharyngitis | 2 |
| *c) P/R + Telaprevir* | | |
|  | Urinary tract infection | 3 |
|  | Abscess due to cat's bite | 3 |
|  | Pneumonia | 3 |
|  | Parotitis | 3 |
|  | Urinary tract infecion | 2 |
|  | Prostatitis | 2 |
|  | Perianal infection | 2 |
|  |  |  |
| **2) Prospective:** | |  |
| *a) Dual therapy with peginterferon /ribavirin (P/R)* | | grade |
|  | no infections |  |
| *b) P/R + Boceprevir* | | |
|  | Enterocolitis | 3 |
|  | Pharyngitis | 3 |
|  | Enterocolitis | 2 |
|  | Otitis | 2 |
| *c) P/R + Telaprevir* | | |
|  | Enterocolitis x3 | 2 |
